# Influence of Maternal Active and Secondhand Smoking during Pregnancy on Childhood Obesity at 3 Years of Age: A Nested Case–Control Study from the Japan Environment and Children’s Study (JECS)

**DOI:** 10.3390/ijerph182312506

**Published:** 2021-11-27

**Authors:** Sayaka Horiuchi, Ryoji Shinohara, Sanae Otawa, Megumi Kushima, Yuka Akiyama, Tadao Ooka, Reiji Kojima, Hiroshi Yokomichi, Kunio Miyake, Hiroyuki Hirai, Koichi Hashimoto, Michio Shimabukuro, Zentaro Yamagata, Japan Environment and Children’s Study Group

**Affiliations:** 1 Center for Birth Cohort Studies, University of Yamanashi, Yamanashi 409-3898, Japan; rshinohara@yamanashi.ac.jp (R.S.); osanae@yamanashi.ac.jp (S.O.); kumegumi@yamanashi.ac.jp (M.K.); zenymgt@yamanashi.ac.jp (Z.Y.); 2 Department of Health Sciences, School of Medicine, University of Yamanashi, Yamanashi 409-3898, Japan; yukaa@yamanashi.ac.jp (Y.A.); tohoka@yamanashi.ac.jp (T.O.); kojimar@yamanashi.ac.jp (R.K.); hyokomichi@yamanashi.ac.jp (H.Y.); kmiyake@yamanashi.ac.jp (K.M.); 3 Department of Diabetes, Endocrinology, and Metabolism, Fukushima Medical University, Fukushima 960-1295, Japan; hiroyuki@fmu.ac.jp (H.H.); mshimabukuro-ur@umin.ac.jp (M.S.); 4 Department of Internal Medicine, Shirakawa Kosei General Hospital, Fukushima 961-0005, Japan; 5 Fukushima Regional Centre for the Japan Environmental and Children’s Study, Fukushima 960-1295, Japan; don@fmu.ac.jp; 6 Department of Paediatrics, School of Medicine, Fukushima Medical University, Fukushima 960-1295, Japan

**Keywords:** tobacco smoking, maternal exposure, secondhand smoking, childhood obesity

## Abstract

Maternal smoking during pregnancy is a risk factor for childhood obesity; however, the combined effect of secondhand smoking during pregnancy on children in the early years is unclear. We examined the effects of maternal active and secondhand smoking during pregnancy on childhood obesity in a large population-based cohort. A nested case–control study originating from the Japan Environment and Children’s Study was performed. The maternal smoking status was collected via self-administered questionnaires during mid/late pregnancy. Obesity in children was determined based on BMI measured at 3 years of age. In total, 4875 cases and 19,491 controls were included in the analyses. Conditional logistic regression models with a significance level of 5% (two-tailed test) were used to test the association. The proportion of mothers who continued smoking and who were exposed to secondhand smoking daily during pregnancy were 3.9% and 13.0% in cases and 2.9% and 10.8% in controls, respectively. Continuous maternal smoking was associated with increased odds of obesity compared to those who never smoked or quit smoking before the pregnancy (adjusted odds ratio, 1.39; 95% confidence interval, 1.01–1.92). The odds increased further when combined with secondhand smoking. The promotion of non-smoking among family members, in public and workplace could benefit pregnant women and offspring.

## 1. Introduction

The prevalence of overweight or obesity in children under 5 years of age has increased dramatically in the last two decades worldwide, from 32 million in 1990 to 38 million in 2019 [1]. It is an important public health concern to prevent childhood obesity as it can increase the risk of various disorders, such as diabetes, cardiovascular diseases, and respiratory diseases in the later part of life [2].

Maternal smoking during pregnancy has been reported as a risk factor for childhood obesity [3,4,5]. Children born to mothers who smoke were found to become obese by 2 years of age at the earliest among low-income populations in the United States [6]. Overweight at 3 years of age was also reported among children whose mothers smoked during early pregnancy among low-income and disadvantaged populations in the United States [7,8]. In Japan, it has been reported that maternal smoking is associated with childhood obesity at 5 years of age, and that the effect persists until 9−10 years of age [9,10,11]. 

Prenatal exposure to secondhand smoke is also reported as a risk factor of childhood obesity [12]; however, it is not clear what is the effect of the combination of maternal active smoking and secondhand smoking on childhood obesity. There was a decline in the maternal smoking rate during pregnancy in Japan to 2.7% in 2017, owing to the success of various public health interventions [13]. In contrast, exposure to secondhand smoke remains high. The International Tobacco Control (ITC) Japan Wave 1 Survey (Feb–Mar 2018) reported the smoking prevalence rate in workplaces, restaurants, and bars as 49%, 55%, and 83%, respectively [14]. Therefore, it is important to understand the significance of secondhand smoke on child health.

The present study aims to investigate (i) whether maternal active and secondhand smoking during pregnancy increases the offspring’s risk of obesity at 3 years of age in the Japanese population and (ii) how effects of maternal active and secondhand smoking interact with each other.

## 2. Materials and Methods 

### 2.1. Study Setting and Population

The present study was a nested case–control study originating from the Japan Environment and Children’s Study (JECS), which is an ongoing nationwide prospective birth cohort study launched in January 2011. Its detailed protocols have been published elsewhere [15,16]. Between 2011 and 2014, 15 Regional Centres across the nation recruited pregnant women who resided in the catchment areas of the centres, had expected due dates between 1 August 2011 and mid-2014 and had no difficulty in understanding Japanese [15]. Their partners and offspring were also recruited and followed-up by the Regional Centres. Assessments at baseline and 1 year after study inception showed representativeness of the study population to the general Japanese population [17,18].

Among the participants of the JECS, only singleton children were included in the present study, as singleton babies were considered to have different birth weights and growth patterns after birth compared to multiple-birth babies [19,20]. Children who had their heights and weights measured at 3 years of age (33 to < 39 months) were included in the analyses. A large number of study participants (*n* = 30,548, 31.0%) did not have their anthropometry measurements recorded at proper timing and had to be excluded from the study. We performed a nested case–control study to improve comparability between two groups in response to a large number of participants who did not have their anthropometry measured. Each case (defined as obesity at 3 years of age determined as >95 percentile in Body Mass Index: BMI) was frequently matched with four controls (defined as non-obesity at 3 years of age) by gestational week, birth weight, and maternal age without replacement. Caliper width for gestational week, birth weight, and maternal age was set as 1 week, 300 g, and 5 years, respectively.

### 2.2. Measure of Exposure

The information on the smoking status of the parents and secondhand smoke exposure was collected using self-administered questionnaires at early and mid/late pregnancy. The present study used the information collected at mid/late pregnancy to ensure capturing smoking status from conception to the time of responding to the questionnaires. The questionnaires were mailed or handed out by co-operating health care providers to each woman and were submitted to the Regional Centres. The expectant mothers were asked to choose whether they or their partners had ever smoked, quit smoking before pregnancy, quit smoking after pregnancy, or were still smoking. For secondhand smoking, the expectant mothers answered how often they inhaled tobacco smoke at home, workplace, or any other indoor places before and during pregnancy, respectively, by choosing frequency from either ‘seldom’, ‘one day per week’, ‘two to three days per week’, ‘four to six days per week’ or ‘every day’. ‘One day per week’ to ‘four to six days per week’ were re-categorised as ‘several times per week’. We also created a variable that was a combination of maternal active and secondhand smoking during pregnancy.

### 2.3. Outcome Definitions and Measurements

The primary outcome was childhood obesity at 3 years of age. A child was considered to be obese when their BMI was >95 percentile according to the child growth standards of the World Health Organization (WHO) according to sex [21]. We decided to use the WHO growth chart to maintain comparability across different countries by using the global standard. The BMI was calculated based on the height and weight reported via self-administered questionnaires. To minimise measurement errors, caregivers were asked to report the most recent height and weight that were measured at well-child visits or at hospitals and mention the date of measurement, if available. If unavailable, height and weight measured at nursery schools or homes were entered. Only birth weight was extracted from medical record transcripts from the co-operating healthcare providers. 

### 2.4. Measure of Covariates

Maternal body weight as well as the health-related behaviours of mothers, such as alcohol and food consumption, were considered as maternal factors that influence the body composition of offspring [22,23,24,25,26,27]. Consumption of Omega-3 fatty acid that is included in red-fish during pregnancy can reduce risk of low birth weight [27] and can lower the risk of obesity at a later time in life. Therefore, we included frequency of red-fish consumption between conception to mid/late pregnancy based on self-reported Food Frequency Questionnaire. Socioeconomic status was also reported to be associated with obesity and considered in this study [28,29,30]. Adiposity rebound before 3 years of age was also considered in the present study. Children whose BMI at 3 years of age was greater than BMI at 1.5, 2 or 2.5 years—whichever available—were considered to have rebound adiposity before 3 years of age. Adiposity rebound was first defined by Rolland-Cachera et al. as rapid growth in body fat after the first phase of growth in adipose tissues in the first year of life [31]. The earlier occurrence of adiposity rebound was reported to be a predictor of future obesity in children [32,33]. Overall, sex of the child, adiposity rebound, maternal obesity before pregnancy, maternal job type at conception, maternal educational level, family income, alcohol drinking status of the mother during pregnancy, maternal fish intake during pregnancy, child’s attendance at nursery school by 1 year of age, and breastfeeding were identified as possible confounders. Breastfeeding was categorized into (1) exclusive breastfeeding, (2) mixed (combination of breastmilk and formula milk), and (3) formula milk only, for up to 6 months of age. Child sex, maternal height and weight before pregnancy were recorded at the co-operating healthcare provider where the mother gave birth. Maternal BMI was categorised into four groups: <18.5 kg/m^2^ (underweight), 18.5 to <25 kg/m^2^ (normal), 25 to <30 kg/m^2^ (overweight), and ≥30 kg/m^2^ (obese). All other covariates were collected from mothers or caregivers via self-administered questionnaires at entry, mid/late pregnancy, 1 month, 6 months, and 1 year after childbirth.

### 2.5. Statistical Analyses

The dataset of jecs-ta-20190930 was used for analyses. First, the distributions and frequency of exposures, outcomes, and covariates according to cases and controls were analysed and summarised as proportions (percentages) or means as appropriate. Univariate analyses were then performed to assess the association of obesity at 3 years of age to maternal smoking during pregnancy and other covariates, respectively, using a conditional logistic regression model. Multivariate analyses were performed by adding all covariates to evaluate the associations between maternal smoking during pregnancy and child obesity using a conditional logistic regression model. We selected covariates on a priori hypothesis basis, and included all the covariates in the multivariate model unless there was evidence of multicollinearity. The interaction between sex of the child and maternal smoking on obesity was further examined by using the likelihood ratio test (LRT), as it was previously reported that the risk of childhood obesity might differ by sex [10]. Finally, we examined the association between the combination of maternal active and secondhand smoking during pregnancy and childhood obesity. LRT was applied to examine multiplicative and additive interaction between maternal active and secondhand smoking. A significance level of 5% with a two-tailed test was applied for all statistical tests. All analyses were performed using STATA/MP 16.1.

### 2.6. Ethics Approval

This study complied with the World Medical Association Declaration of Helsinki and Ethical Guidelines for Medical and Health Research involving Human Subjects that was promulgated by the Ministry of Health, Labour and Welfare, Japan. The JECS protocol was approved by the Ministry of the Environment’s Institutional Review Board on Epidemiological Studies (no. 100406001) and approved by the ethics committees of all participating institutions. Written informed consent was obtained from all participants.

## 3. Results

**Figure 1:**
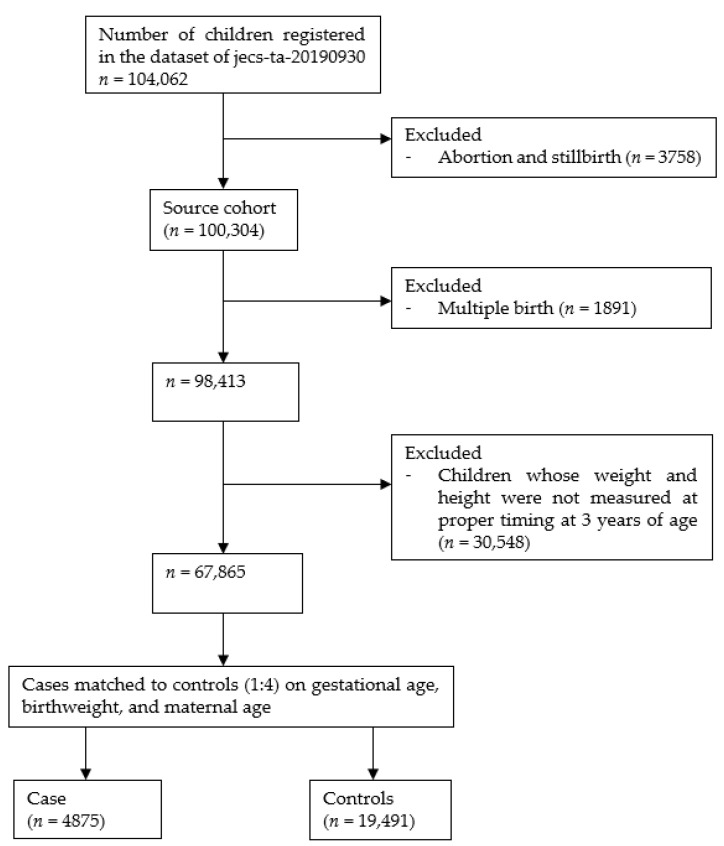
Selection of study participants.

Among the 100,304 registered live births, multiple births (*n* = 1891) were excluded. Among 98,413 children, 30,548 who did not have their weights and heights measured at 3 years of age (33 to < 39 months) were excluded (Figure 1). Among those that were excluded, the proportions of children exposed to tobacco smoke and those at a lower socioeconomic status were higher compared to those that were included (Table 1). In total, 4875 cases and 19,491 controls were identified and included in the analyses. Among them, 12.6% of cases and 14.6% of controls had their weights and heights measured at well-child visits or at hospitals. The majority had undergone anthropometric measurements at nursery schools (58.5% of cases and 55.9% of controls). The characteristics of the study participants are summarised in Table 2. The proportion of mothers and fathers who continued smoking during pregnancy was 3.9% and 46.1% of cases, respectively, and 2.9% and 42.8% of controls, respectively. Mothers who were exposed to secondhand smoking every day during pregnancy accounted for 13.0% of cases and 10.8% of controls. Almost half of the children were male in both cases (52.6%) and controls (52.9%). More than half of the mothers in both groups were employed. Maternal obesity (BMI ≥ 30 kg/m^2^) was 4.7% in the cases and 2.2% in the controls. There were significant differences in birth weight and gestational week at birth between cases and controls (*p* < 0.001).

Table 3 summarises the results of univariate and multivariate analyses on the association between childhood obesity and maternal active smoking during pregnancy, and childhood obesity and other covariates. In univariate analyses, children had higher odds of being obese at 3 years of age when their mothers quit smoking after pregnancy (odds ratio (OR), 1.22; 95% confidence interval (CI), 1.11–1.35) or continued smoking during pregnancy (OR, 1.40; 95% CI, 1.18–1.65) compared to those whose mothers never smoked or quit smoking before pregnancy. In the multivariate analysis, exposure to secondhand smoking before pregnancy was excluded due to a strong correlation between secondhand smoking before pregnancy and secondhand smoking during pregnancy. After adjusting for covariates, continuous maternal smoking during pregnancy was still associated with an increased risk of obesity (OR, 1.39; 95% CI, 1.01–1.92) relative to mothers who never smoked or quit smoking before pregnancy. Exposure to secondhand smoking during pregnancy was also associated with higher odds of obesity among those who were exposed everyday (OR, 1.23; 95% CI, 1.01–1.50) compared to non-exposure. There was no multiplicative interaction in the effects on childhood obesity between maternal smoking during pregnancy and child sex (LRT: *p* = 0.988), and between maternal smoking and exposure to secondhand smoking during pregnancy (LRT: *p* = 0.142).

Nonetheless, an additive interaction existed between maternal smoking and secondhand smoking (LRT: *p* = 0.033). The odds of obesity tended to be higher when a higher exposure to maternal active and secondhand smoking was combined compared to the group with no maternal active smoking and seldom exposure to secondhand smoking (Table 4).

## 4. Discussion 

The present study showed that continuous maternal smoking during pregnancy was associated with an increased risk of childhood obesity at 3 years of age in the Japanese population, which has a relatively low prevalence of obesity compared to the global average [34,35]. The result was consistent with those of previous studies that showed an association between maternal smoking and childhood obesity in other countries [3,4,9,36]. It was believed that increased concentrations of nicotine and carbon monoxide in the uterus increase the risk of intrauterine growth retardation and a low birth weight [37,38]. Then, slow growth during the foetal and infant period could lead to a rapid weight gain and obesity in childhood [39]. Contrastingly, this study showed that maternal smoking could increase the risk of obesity as early as 3 years irrespective of birthweight. The independent effect of maternal smoking from birthweight in this study underscored the epigenetic effect of maternal smoking on obesity. Dissimilar to several studies that reported sex-related differences in the likelihood of obesity [9,40], there was no difference in the effects of maternal smoking during pregnancy on childhood obesity by children’s sex in the present study. 

The present study also showed that there was additive interaction between maternal active and secondhand smoking, and the odds of obesity further increased when both the exposures were combined: high frequent exposure to both maternal active and secondhand smoking had higher odds of obesity. The odds of childhood obesity associated with secondhand smoking were lower than those associated with maternal smoking during pregnancy, suggesting that maternal smoking might have a greater impact on childhood adiposity due to intrauterine influence [41]. However, the impact of secondhand smoking on child health could be tremendous given its high proportion among pregnant women. These results suggest the importance of adopting measures to simultaneously prevent both maternal active and secondhand smoking to reduce childhood obesity.

The high smoking prevalence among expectant fathers in Japan (>40%) is also concerning [13]; however, its association with obesity disappeared after adjusting for secondhand smoking. Fathers may have avoided smoking at home, but we are unable to know this fact because the place of smoking was not asked in the study. 

The present study had several limitations. First, the smoking status of the parents was self-reported; therefore, there might have been underreporting [42,43]. Nonetheless, the smoking status was asked before the outcome was measured. Therefore, the underreporting occurred non-differentially as per the outcome status, and this might have led to an underestimation of the effects of smoking on childhood obesity. Therefore, we believe that underreporting did not influence our conclusions. Second, the intensity of smoking, such as the quantification of cigarette consumption per day, was not measured; therefore, the exposure intensity might differ even among mothers who continued to smoke during pregnancy. There might have been variation in the level of tobacco exposure within the same exposure group in this study. This also applies to the level of exposure to secondhand smoking, as the average time duration of exposure per day was not investigated. Thirdly, only one fifths of participants had undergone anthropometric measurements at well-child visits or hospitals; therefore, there might have been measurement errors. However, there was not a large difference in the distribution of the place of measurement between cases and controls, and the measurement error may have arisen non-differentially. We, therefore, believe that the measurement error did not influence our conclusions. Fourth, a large amount of the study population was excluded due to the lack of anthropometric data. The excluded had a higher prevalence of smoking as well as maternal obesity and were at a lower socioeconomic status compared to those included. Therefore, the study population was biased towards people at less risk of childhood obesity. Finally, the present study did not consider environmental factors after birth such as eating habits during early childhood. It has been reported that children born to mothers who smoke tend to consume more calories [6,44], and nutrition during early life may be related to rapid growth in body weight in early life. However, considering the purpose of the present study was to examine effects of prenatal exposure to tobacco smoke from perspectives of epigenetics, the exclusion of eating habits after birth would be reasonable.

These limitations did not change the interpretation of the association between maternal active and secondhand smoking and childhood obesity in the present study. It is necessary to support cigarette cessation before and during pregnancy to prevent childhood obesity. Measures to prevent secondhand smoking in public and workplaces should also be intensified.

## 5. Conclusions

Our findings suggested that continuous maternal smoking during pregnancy and exposure to secondhand smoking can increase the risk of childhood obesity at 3 years of age in Japan. It is necessary to support both mothers and family members with cigarette cessation before and during pregnancy to prevent childhood obesity. Measures to prevent secondhand smoking in public and workplaces should also be intensified.

## Figures and Tables

**Table 1:** Comparison of characteristics of children whose heights and weights were measured and not measured at 3 years of age.

Variable	Not Measured (*n* = 30,548)	Measured (*n* = 67,865)	*p*-Value (chi-Square)
Maternal smoking during pregnancy			
Never or quit smoking before pregnancy	21,300 (69.7)	57,039 (84.1)	<0.001
Quit smoking after pregnancy	5249 (17.2)	7885 (11.6)	
Continue smoking	2241 (7.3)	2180 (3.2)	
Missing data	1758 (5.8)	761 (1.1)	
Paternal smoking during pregnancy			
Never or quit smoking before pregnancy	12,079 (39.5)	35,708 (52.6)	<0.001
Quit smoking after pregnancy	872 (2.9)	1894 (2.8)	
Continue smoking	15,368 (50.3)	28,974 (42.7)	
Missing data	2229 (7.3)	1289 (1.9)	
Exposure to secondhand smoking before pregnancy			
Rare	12,865 (42.1)	35,384 (52.1)	<0.001
Several times per week	9435 (30.9)	21,360 (31.5)	
Everyday	7077 (23.2)	10,547 (15.5)	
Missing data	1171 (3.8)	574 (0.9)	
Exposure to secondhand smoking during pregnancy			
Rare	16,207 (53.1)	43,748 (64.5)	<0.001
Several times per week	7622 (25.0)	16,403 (24.2)	
Everyday	5233 (17.1)	7202 (10.6)	
Missing data	1486 (4.9)	512 (0.8)	
Child sex			
Male	15,740 (51.5)	34,694 (51.1)	
Female	14,790 (48.4)	33,171 (48.9)	
Unknown	3 (0.01)	-	
Missing	15 (0.1)	-	
Birthweight (g)	2997.8 [469.2]	3034.5 [394.9]	<0.001 *
Gestational week at birth (week)	38.6 [2.0]	38.9 [1.4]	<0.001 *
Breastfeeding during first 6 months			
Exclusive	8257 (27.0)	24,935 (36.7)	<0.001
Mixed	15,594 (51.1)	40,841 (60.2)	
Formula	808 (2.7)	1275 (1.9)	
Missing data	5889 (19.3)	814 (1.2)	
Attendance to nursery school by 1 year of age			
Yes	5372 (17.6)	18,382 (27.1)	<0.001
No	16,720 (54.7)	47,973 (70.7)	
Missing data	8456 (27.7)	1510 (2.2)	
Maternal age at entry (year)	29.8 [5.3]	31.2 [4.9]	<0.001 *
Maternal job type at conception			
Employed	17,392 (56.9)	43,743 (64.5)	<0.001
Housewife	8974 (29.4)	18,777 (27.7)	
Students, unemployed or others	2121 (6.9)	3367 (5.0)	
Missing data	2061 (6.8)	1978 (2.9)	
Maternal educational attainment			
Elementary to junior high-school	13,766 (45.1)	22,756 (33.5)	<0.001
Under-graduate	14,876 (48.7)	43,341 (63.9)	
Post-graduate	279 (0.9)	1131 (1.7)	
Missing data	1627 (5.3)	637 (0.9)	
Income (million yen)			
<4	12,306 (40.3)	23,843 (35.1)	<0.001
4–6	8242 (27.0)	21,430 (31.6)	
6–8	3463 (11.3)	10,832 (16.0)	
≥8	2310 (7.6)	7384 (10.9)	
Missing data	4227 (13.8)	4376 (6.5)	
Marital status at registration			
Married	27,627 (90.4)	64,892 (95.6)	<0.001
Single, divorced or widowed	1802 (5.9)	2408 (3.6)	
Missing data	1119 (3.7)	565 (0.8)	
Maternal BMI before pregnancy (kg/m^2^)	21.5 [3.6]	21.1 [3.2]	
<18.5 (underweight)	5002 (16.4)	10,911 (16.1)	<0.001
18.5–25 (normal)	21,530 (70.5)	50,371 (74.2)	
25–30 (overweight)	2912 (9.5)	5076 (7.5)	
≥30 (obesity)	1017 (3.3)	1465 (2.2)	
Missing data	87 (0.3)	42 (0.1)	
Maternal drinking during pregnancy			
No	9512 (31.1)	22,656 (33.4)	<0.001
Quit	18,415 (60.3)	42,610 (62.8)	
Continue	904 (3.0)	1802 (2.7)	
Missing data	1717 (5.6)	797 (1.2)	
Maternal diabetes or gestational diabetes			
No	29,291 (95.9)	65,756 (96.9)	0.448
Yes	968 (3.2)	2109 (3.1)	
Missing data	289 (0.9)	-	

Values are number (%) or mean [SD]; SD, standard deviation; BMI, body mass index; * *t*-test was applied.

**Table 2:** Characteristics of the study participants (*n* = 24,366).

Variable	Cases (*n* = 4875)	Controls (*n* = 19,491)
Maternal smoking during pregnancy		
Never or quit smoking before pregnancy	3971 (81.5)	16,458 (84.4)
Quit smoking after pregnancy	669 (13.7)	2290 (11.8)
Continue smoking	188 (3.9)	565 (2.9)
Missing data	47 (1.0)	178 (0.9)
Paternal smoking during pregnancy		
Never or quit smoking before pregnancy	2417 (49.6)	10,260 (52.6)
Quit smoking after pregnancy	128 (2.6)	550 (2.8)
Continue smoking	2245 (46.1)	8338 (42.8)
Missing data	85 (1.7)	343 (1.8)
Exposure to secondhand smoking before pregnancy		
Rare	2385 (48.9)	10,250 (52.6)
Several times per week	1601 (32.8)	6188 (31.8)
Everyday	875 (18.0)	2988 (15.3)
Missing data	14 (0.3)	65 (0.3)
Exposure to secondhand smoking during pregnancy		
Rare	2942 (60.4)	12,587 (64.6)
Several times per week	1272 (26.1)	4677 (24.0)
Everyday	633 (13.0)	2119 (10.8)
Missing data	28 (0.6)	108 (0.6)
Child sex		
Male	2562 (52.6)	10,309 (52.9)
Female	2313 (47.5)	9182 (47.1)
Birthweight (g) ^1^	3182.5 [391.8]	3123.6 [389.0]
Gestational week at birth (week) ^1^	38.9 [1.4]	39.0 [1.3]
Breastfeeding during first 6 months		
Exclusive	1689 (34.7)	7238 (37.1)
Mixed	3006 (61.7)	11,662 (59.8)
Formula	124 (2.5)	365 (1.9)
Missing data	56 (1.2)	226 (1.2)
Attendance to nursery school by 1 year of age		
Yes	1441 (29.6)	5290 (27.1)
No	3316 (68.0)	13,780 (70.7)
Missing data	118 (2.4)	421 (2.2)
Adiposity rebound before 3 years of age		
No	2030 (41.6)	13,242 (67.9)
Yes	2645 (54.3)	5519 (28.3)
Missing data	200 (4.1)	730 (3.8)
Maternal age at entry (year) ^1^	31.1 [4.9]	31.2 [4.8]
Maternal job type at conception		
Employed	3213 (65.9)	12,633 (64.8)
Housewife	1305 (26.8)	5530 (28.4)
Students, unemployed or others	225 (4.6)	893 (4.6)
Missing data	132 (2.7)	435 (2.2)
Maternal educational level		
Elementary to junior high-school	1772 (36.4)	6506 (33.4)
Under-graduate	2984 (61.2)	12,501 (64.1)
Post-graduate	81 (1.7)	341 (1.8)
Missing data	38 (0.8)	143 (0.7)
Income (million yen)		
<4	1743 (35.8)	6831 (35.1)
4–6	1513 (31.0)	6145 (31.5)
6–8	784 (16.1)	3123 (16.0)
≥8	519 (10.6)	2177 (11.0)
Missing data	316 (6.5)	1215 (6.2)
Maternal BMI before pregnancy (kg/m^2^)	22.2 (3.8)	21.2 (3.2)
<18.5 (underweight)	434 (8.9)	3002 (15.4)
18.5–25 (normal)	3629 (74.4)	14,541 (74.6)
25–30 (overweight)	583 (12.0)	1511 (7.8)
≥30 (obesity)	227 (4.7)	431 (2.2)
Missing data	2 (0.1)	6 (0.1)
Maternal drinking during pregnancy		
No	1584 (32.5)	6499 (33.3)
Quit	3131 (64.2)	12,260 (62.9)
Continue	121 (2.5)	535 (2.7)
Missing data	39 (0.8)	197 (1.0)
Maternal fish intake during pregnancy		
Less than once a month	1005 (20.6)	3827 (19.6)
Several times a month	1071 (22.0)	4663 (23.9)
Once a week	1186 (24.3)	4781 (24.5)
More than once a week	1559 (32.0)	6014 (30.9)
Missing data	54 (1.1)	206 (1.1)

Values are number (%) or mean [SD]; SD, standard deviation; BMI, body mass index. ^1^ *p*-values of *t*-tests to compare means between cases and controls produced were <0.001 for birth weight and gestational week at birth, and 0.228 for maternal age at entry.

**Table 3:** Odds ratios of being obese at 3 years of age in relation with maternal smoking during pregnancy and other variables with conditional logistic regression models (*n* = 24,366).

Smoking Status during Pregnancy	Crude OR (95% CI)	Adjusted OR ^a^ (95% CI) *n* = 17,788
Maternal smoking during pregnancy		
Never or quit smoking before pregnancy	1.00	1.00
Quit smoking after pregnancy	1.22 (1.11–1.35)	1.14 (0.95–1.37)
Continue smoking	1.40 (1.18–1.65)	1.39 (1.01–1.92)
Paternal smoking during pregnancy		
Never or quit smoking before pregnancy	1.00	1.00
Quit smoking after pregnancy	0.98 (0.81–1.20)	0.86 (0.61–1.21)
Continue smoking	1.15 (1.08–1.23)	1.03 (0.91–1.17)
Exposure to secondhand smoking before pregnancy		
Seldom	1.00	- ^b^
Several times per week	1.12 (1.04–1.20)	-
Everyday	1.27 (1.16–1.38)	-
Exposure to secondhand smoking during pregnancy		
Seldom	1.00	1.00
Several times per week	1.17 (1.09–1.26)	1.14 (0.99–1.30)
Everyday	1.29 (1.17–1.42)	1.23 (1.01–1.50)

BMI, body mass index; OR, odds ratios; CI, confidence interval. ^a^ Adjusted for all other variables in the table, child sex, breastfeeding, attendance to nursery school by 1 year of age, adiposity rebound before 3 years of age, maternal job type at conception, maternal educational level, income, maternal BMI before pregnancy (kg/m^2^), maternal drinking during pregnancy, maternal fish intake during pregnancy, birthweight (continuous variable), maternal age at entry (continuous variable) and gestational week at birth (continuous variable). ^b^ Omitted from the multivariable analysis due to collinearity with exposure to secondhand smoking during pregnancy.

**Table 4:** Odds ratios of being obese at 3 years of age in relation with combination of maternal active and secondhand smoking during pregnancy (*n* = 17,824).

Maternal Smoking during Pregnancy	Exposure to Secondhand Smoking during Pregnancy		
	Seldom	Several Times per Week	Everyday
Never or quit smoking before pregnancy	1.00	1.11 (0.97–1.27)	1.23 (0.98–1.55)
Quit smoking after pregnancy	1.11 (0.97–1.27)	1.30 (0.98–1.73)	1.59 (1.17–2.15)
Continue smoking	1.23 (0.98–1.55)	1.59 (1.17–2.15)	1.60 (1.06–2.39)

Values are odds ratios (95% confidence intervals). Adjusted for paternal smoking during pregnancy, child sex, breastfeeding, attendance to nursery school by 1 year of age, adiposity rebound before 3 years of age, maternal job type at conception, maternal educational level, income, maternal BMI before pregnancy (kg/m^2^), maternal drinking during pregnancy, maternal fish intake during pregnancy, birthweight (continuous variable), maternal age at entry (continuous variable) and gestational week at birth (continuous variable).

## Data Availability

The data were unsuitable for public deposition due to ethical restrictions and the legal framework of Japan. It is prohibited by the Act on the Protection of Personal Information (Act no. 57 of 30 May 2003, amended on 9 September 2015) to publicly deposit data containing personal information. Ethical Guidelines for Medical and Health Research Involving Human Subjects enforced by the Japan Ministry of Education, Culture, Sports, Science and Technology, and the Ministry of Health, Labour and Welfare also restrict the open sharing of epidemiologic data. All inquiries about access to data should be sent to jecs-en@nies.go.jp. Shoji F. Nakayama, JECS Programme Office, National Institute for Environmental Studies, is responsible for handling inquiries sent to this e-mail address.
